# On the superiority of a combination of aerobic and resistance exercise for fibromyalgia syndrome: A network meta-analysis

**DOI:** 10.3389/fpsyg.2022.949256

**Published:** 2022-09-28

**Authors:** Jiping Chen, Bing Han, Chenggang Wu

**Affiliations:** ^1^School of Physical Education, Shandong University, Jinan, China; ^2^Key Laboratory of Multilingual Education With AI, School of Education, Shanghai International Studies University, Shanghai, China; ^3^Institute of Linguistics, Shanghai International Studies University, Shanghai, China

**Keywords:** fibromyalgia syndrome, quality of life, pain, physical function, aerobic exercise, resistance training, combined exercise

## Abstract

**Background:**

Fibromyalgia syndrome is the second most common chronic diffuse pain disorder and can have a lasting negative impact on the quality of life, pain, and physical function of people. Exercise therapy is an important component of the treatment of fibromyalgia, but there was not a consensus understanding of the effect of various exercise programs on the quality of life, pain, and physical function of people with fibromyalgia syndrome. This study aimed to compare three exercise programs (aerobic exercise, resistance exercise, and a combination of aerobic and resistance exercise) in their effectiveness in improving quality of life, relieving muscle pain, and enhancing physical function in patients with fibromyalgia.

**Methods:**

A comprehensive search of databases, including China National Knowledge Internet, Wan fang, The Cochrane Library, PubMed, EMBASE, and Web of Science, was conducted to identify randomized controlled trials on exercise therapy for patients with fibromyalgia syndrome with outcome indicators including at least one of Fibromyalgia Impact Questionnaire (FIQ), Tender point count (TPC), and 6-minute walk test (6MWT) from the date of database creation on 20 April 2022. The included studies were evaluated for literature quality according to Cochrane Handbook criteria, and a network meta-analysis was performed using STATA 14.0.

**Result:**

Forty-five randomized controlled trials met all inclusion criteria and were analyzed. The network meta-analysis showed that a combination of aerobic and resistance exercise was ranked first in all three dimensions of quality of life improvement, pain alleviation, and physical function enhancement (Mean Rank = 1.6, 1.2, 5.9).

**Conclusion:**

The current meta-analysis demonstrates that the combination of aerobic and resistance exercise may be the best type of exercise to accentuate the quality of life, pain alleviation, and physical function for people with fibromyalgia syndrome.

## Introduction

Fibromyalgia Syndrome (FMS) is a type of chronic diffuse pain that causes widespread discomfort, exhaustion, sleep difficulties (Yunus and Aldag, 1996), cognitive impairment (Alanoglu et al., 2005), depression, and anxiety, among other aspects. FMS also has a negative impact on patients with fibromyalgia syndrome's quality of life, muscle discomfort (Arnold et al., 2019), and physical function (Mannerkorpi et al., 1994). FMS has a global incidence rate of 2–4%, with women (4.2%) being more affected than men (0.2%) (Mas et al., 2008). Following low back pain and osteoarthritis, it is the second most common musculoskeletal condition (Häuser et al., 2015). FMS is, moreover, becoming more common as the population ages and unhealthy behaviors become more prevalent in today's society (Shillam et al., 2011). As a result, there is a pressing need for simple, effective, short-term, cost-effective, and safe therapeutic solutions.

FMS has an unknown origin and pathology but it may entail central sensitization (Häuser et al., 2015), aberrant neurotransmitter production (Sarzi-Puttini et al., 2020), immunological diseases, and abnormal neurological functioning (Ji et al., 2018). As a result, both patients and healthcare providers face difficulties in diagnosing and treating FMS (Macfarlane et al., 2017). FMS can be treated by using pharmacological and non-pharmacological therapy to reduce pain, mood changes, and exhaustion in fibromyalgia sufferers (Seto et al., 2019). For example, commonly used pharmacological therapies include pregabalin, norepinephrine reuptake inhibitors, amitriptyline, etc. Non-pharmacological therapies include health education, exercise, cognitive behavioral therapy, etc (Clauw, 2014). Most medications can only relieve one or two of the symptoms and no medication has been effective in controlling all the symptoms of fibromyalgia patients (Carta et al., 2013; Giacomelli et al., 2013). In this regard, exercise programs appears to be an effective component of treatment, improving pain alleviation and physical function and reducing the burden of FM on quality of life (Jones et al., 2006; Busch et al., 2007). Exercise program has been proven in many clinical practices to be an effective treatment for fibromyalgia and has played a significant role in the treatment of FMS and has been recommended by several FMS management guides (Häuser et al., 2017; Macfarlane et al., 2017).

Common types of exercise programs are aerobic exercise, resistance exercise, and a combination of aerobic and resistance exercise, and all three therapies have been shown to be effective in decreasing the negative symptoms of patients with existing fibromyalgia. For example, aerobic exercise can improve the quality of life and relieve muscle pain in FMS (Busch et al., 2007), while resistance exercise can significantly improve physical function in FMS (Busch et al., 2013). However, most current clinical studies only analyze the efficacy of two exercise programs by comparing each other (Rooks et al., 2007; Alentorn-Geli et al., 2008), and most meta-analyses start with only a few outcome indicators, for example, quality of life, fatigue, and sleep quality (Estévez-López et al., 2021; Albuquerque et al., 2022). The extant studies lack a comparison of the three common exercise programs from the three perspectives of improving quality of life, relieving muscle pain, and simultaneously enhancing physical function in patients with fibromyalgia.

Therefore, the primary aim of this study was to compare the effectiveness of three types of commonly used exercise programs in the treatment of fibromyalgia, choosing the quality of life, muscle pain, and physical function as outcome indicators. In this way, it is intended to identify the most appropriate type of exercise to improve quality of life, reduce muscle pain, and increase physical function in patients with fibromyalgia.

## Methods

This systematic review and network meta-analysis were reported in accordance with the Preferred Reporting Items for Systematic Reviews and Meta-Analyses (PRISMA) statement (Page et al., 2021). This review was registered with the international prospective register of systematic reviews PROSPERO (registration no. CRD42022335679) https://www.crd.york.ac.uk/prospero/display_record.php?RecordID=335679.

### Search strategy

The search was conducted using a combination of each keyword under four topics as search terms: “fibromyalgia syndrome, aerobic exercise, resistance exercise, and combined exercise.” Search databases included China National Knowledge Internet, Wan fang, Database, The Cochrane Library, PubMed, EMBASE, Web of Science, and Medline. The study used the Patients-Intervention-Comparisons-Outcomes-Study (PICOS) as the criteria for inclusion in the literature: (P) Patients: patients with a clear diagnosis of FMS, which should be in accordance with the relevant diagnostic criteria of the American College of Rheumatology (ACR); (I) Intervention: aerobic exercise, resistance exercise, and aerobic combined with resistance exercise; (C): Comparisons: other exercise modality or no exercise control; (O) Outcomes: Fibromyalgia Impact Questionnaire (FIQ) as an outcome indicator of quality of life; Tender point count (TPC) as an outcome indicator of pain; Six-minute walk test (6WMT) as an outcome indicator of physical function; (S) Study: Randomized controlled trials (RCTs).

### Eligibility criteria

Includes randomized controlled trials (excluding conference abstracts, reports, and papers) published in scientific peer-reviewed papers published to date (20 April 2022). Exclusion criteria: (1) non-randomized controlled trials, case reports, the experience of doctors, book reports, own before-and-after controls, review literature; (2) animal studies; (3) purely descriptive studies; (4) repeated published studies; (5) literature with an unclear diagnosis of FMS or combination of other diseases; (6) literature with unclear results, incomplete data or unsuccessful contact with full-text authors. Outcomes of interest are all recommended by the American College of Rheumatology for measurement and are among the most used today include: (1) Fibromyalgia Impact Questionnaire (FIQ); (2) Tender point count (TPC); (3) Six-minute walk test (6WMT).

### Exercise categories

Eight categories along with a control group were used in this study to classify the exercise interventions for the included Randomized controlled trials:

1. Aerobic; Moderate intensity; Short cycle: AE-M-S.
2. Aerobic; Low intensity; Short cycle: AE-L-S.
3. Aerobic; Low intensity; Long cycle: AE-L-L.
4. Resistance; Low intensity; Short cycle: RE-L-S.
5. Resistance; Moderate intensity; Short cycle: RE-M-S.
6. Combined; Low intensity; Short cycle: COM-L-L.
7. Combined; Moderate intensity; Short cycle: COM-M-S.
8. Combined; Moderate intensity; Long cycle: COM-M-L.
9. No exercise; Control group: CON.

Each category was designed using the Frequency, Intensity, Time, and Type (F.I.T.T) principles of exercise prescription and ACSM estimates of cardiorespiratory and resistance exercise intensity (Thompson et al., 2013). A detailed definition of each exercise category is provided in Table 1.

**Table 1:** Definition of the exercise interventions using the F.I.T.T. principle.

**Type of exercise**	**Abbreviation**	**Definition**
Aerobic; Moderate intensity; Short cycle	AE-M-S	Frequency: 1–3 times per week Intensity: >64% HRmax and <75% HRmax Time: Each session lasting 30–60 min; total intervention cycle <24 weeks Type: Any mode of aerobic only (e.g., walking, running, cycling, and swimming)
Aerobic; Low intensity; Short cycle	AE-L-S	Frequency: 1–3 times per week Intensity: ≤ 64% HRmax Time: Each session lasting 30–60 min; total intervention cycle <24 weeks; Type: Any mode of aerobic only (e.g., walking, running, cycling, and swimming)
Aerobic; Low intensity; Long cycle	AE-L-L	Frequency: 1–3 times per week Time: Each session lasting 30–60 min; total intervention cycle ≥24 weeks; Intensity: ≤ 64% HRmax Type: Any mode of aerobic only (e.g., walking, running, cycling, and swimming)
Resistance; Low intensity; Short cycle	RE-L-S	Frequency: 1–3 times per week Intensity: ≤ 50% (1RM) Time: Each session lasting 30–60 min; total intervention cycle <24 weeks; Type: Any mode of resistance exercise (e.g., free weights, weights machines, and resistance bands)
Resistance; Moderate intensity; Short cycle	RE-M-S	Frequency: 1–3 times per week Intensity: >50% (1rm) and <70%(1rm); Time: Each session lasting 30–60 min; total intervention cycle <24 weeks; Type: Any mode of resistance exercise (e.g., free weights, weights machines, and resistance bands)
Combined; Low intensity; Short cycle	COM-L-S	Frequency: 1–3 times per week Intensity: ≤ 50% (1RM) Time: Each session lasting 30–60 min; total intervention cycle <24 weeks Type: Any mode of resistance exercise (e.g., free weights, weights machines, and resistance bands)
Combined; Moderate intensity; Short cycle	COM-M-S	Frequency: 1–3 times per week Intensity: ≤ 50% (1RM) Time: Each session lasting 30–60 min; total intervention cycle <24 weeks Type: Any mode of resistance exercise (e.g., free weights, weights machines, and resistance bands)
Combined; Moderate intensity; Long cycle	COM-M-L	Frequency: 1–3 times per week Intensity: ≤ 50% (1RM) Time: Each session lasting 30–60 min; total intervention cycle ≥24 weeks Type: Any mode of resistance exercise (e.g., free weights, weights machines, and resistance bands)
Control	CON	No exercise

### Study selection

Zotero literature management software was used to manage literature search records. The selection process consisted of three steps. Firstly, the relevant literature retrieved from each database was imported into the Zotero software to delete duplicates. In the second step, all articles selected from the initial stage were reviewed by two independent reviewers by abstract and assessed for eligibility. Any disagreements were resolved through discussion between reviewers and consultation with a third party on the review panel. Finally, the remaining articles were fully reviewed by the two independent reviewers who reviewed the abstracts using predetermined inclusion criteria.

### Data extraction

Information was extracted during the reading of the full article, which included the first author, year of publication, type of study, basic information about the study sample (sample size, age, gender), the various elements of exercise prescription (type of exercise, intensity, frequency, duration, periodicity) and outcome indicators. The data used for analysis in this study were the values of change before and after the intervention (i.e., the values of change from baseline to post-intervention).

### Risk of bias in individual studies

The risk of bias was assessed using Cochrane Review Manager 5.3. Included studies were assessed for quality on 6 indicators: random allocation method, allocation protocol concealment, blinding, completeness of outcome data, selective reporting of study results, and other sources of bias, with each item subdivided into 3 options: high risk, low risk, and uncertain risk. The risk of bias was interpreted and evaluated for quality based on the description of each of these aspects by the included studies.

### Statistical analysis

Network meta-analysis (NMA) was conducted by combining direct and indirect evidence. Direct evidence was obtained directly from a randomized controlled trial, whereas indirect evidence was obtained through one or more common comparators. For example, in the absence of a randomized controlled trial that compares directly aerobic and resistance exercise, a comparison can be made indirectly if aerobic and resistance exercise are compared with no exercise intervention. The NMA was performed using Stata 14.0. Means and standard deviations were used to compare the effects of different exercise interventions on FM. Random effects network meta-analysis was used to estimate the relative impact on multiple intervention comparisons. Mean differences (MDs) and their associated 95% confidence intervals (CIs) were calculated to estimate effect sizes for fibromyalgia Surface under the cumulative ranking curve (SUCRA) values and cumulative ranking plots were used to calculate the ranking probabilities for each intervention. SUCRA value is the probability of each treatment performing best in the network, with higher values indicating higher ranking probabilities.

## Results

### Literature selection

**Figure 1:**
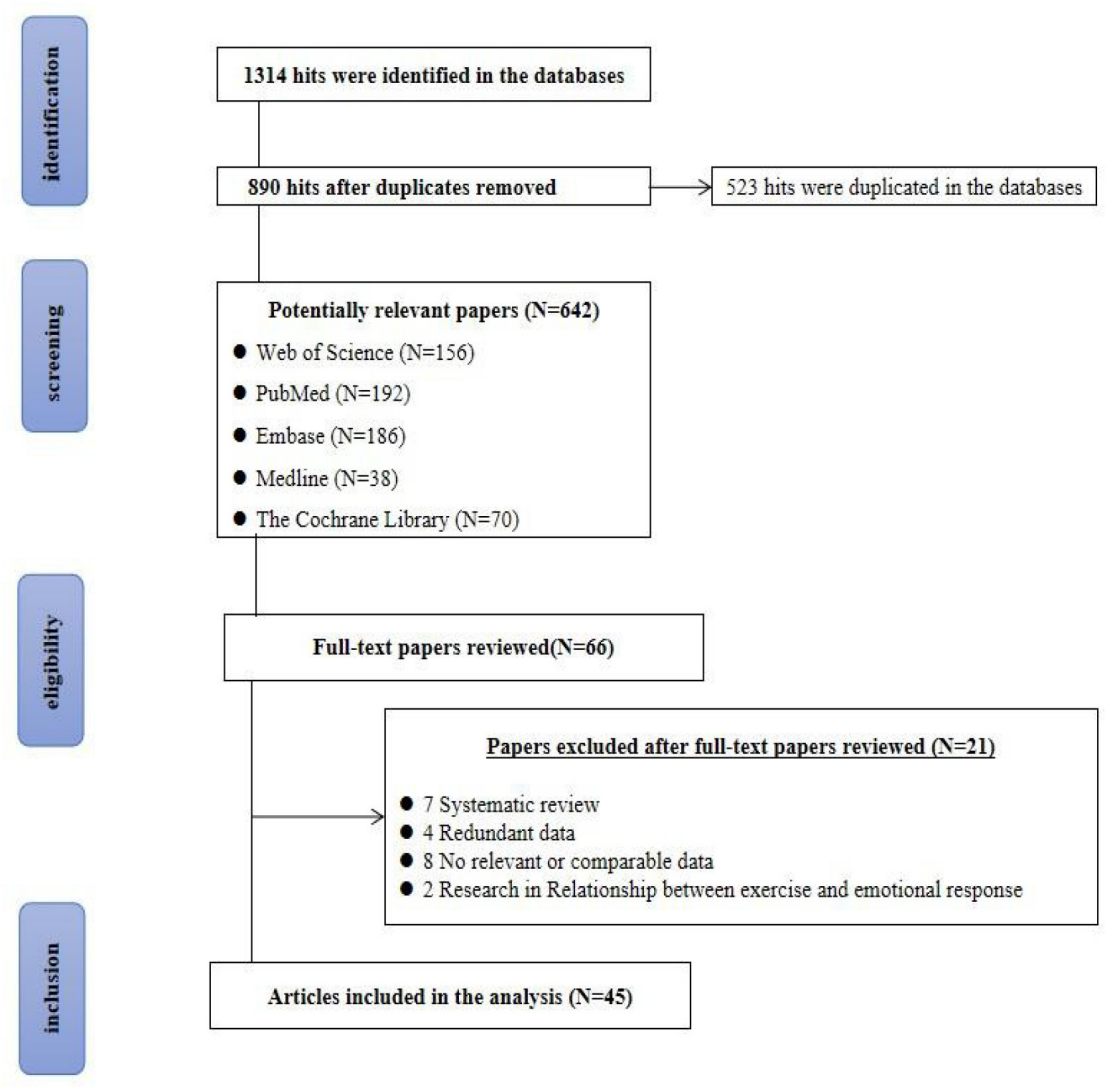
PRISMA flow chart.

Altogether 1,314 articles related to this research topic were retrieved from the database. Then, 890 papers were excluded by Zotero, 642 papers were preliminarily screened after reading the titles and abstracts of the papers, and 66 potential related papers were kept after reading all the first screened papers. Finally, 21 papers that did not meet the inclusion and/or met exclusion criteria of this study were excluded, and 45 papers were finally included in the network meta-analysis. The detailed process is illustrated in Figure 1.

### Characteristics of the included studies

Table 2 displays the characteristics of the included studies. There were 2,904 patients (99.5% females, ages ranging from 30 to 70 years) being examined in 45 studies. Thirty-eight studies included only women and only 7 studies included both women and men.

**Table 2:** Summary of studies included in network meta-analysis indicating the exercise intervention used and the outcome measure.

**No**.	**References**	**Country**	**N(M)**	**Age**	**Period**	**Frequency**	**Intensity**	**Time**	**Type**	**Outcome**
1	Altan et al. (2004)	Turkey	AE-M-S:24(11) CON:22(0)	AE-M-S:43.14 ± 6.39 CON:43.91 ± 6.26	12	3	60% HRmax	35	AE-M-S CON	FIQ Tender point count (1–18)
2	Assumpção et al. (2018)	Brazil	RE-M-S:16(0) CON:14(0)	RE-M-S:45.7 ± 7.7 CON:46.9 ± 6.5	12	2	Borg (13)	40	RE-M-S CON	FIQ Tender point count (1–18)
3	Andrade et al. (2019)	Brazil	RE-M-S:25(0) CON:21(0)	RE-M-S:52.0 ± 9.26 CON: 50.6 ± 9.61	4	3	67% (1rm)	60	RE-M-S CON	FIQ
4	Bircan et al. (2008)	Turkey	RE-M-S:13(0) AE-M-S:13(0)	RE-M-S:46.0 ± 8.5 AE-M-S:48.3 ± 5.3	8	3	65% HRmax	60	RE-M-S AE-M-S	Tender point count (1–18) 6MWT
5	Carbonell-Baeza et al. (2011b)	Spain	COM-M-S:33(0) CON:32(0)	COM-M-S:50.0 ± 7.3 CON:51.4 ± 7.4	16	1	Borg (13)	45	COM-M-S CON	Tender point count (1–18) 6MWT
6	Carbonell-Baeza et al. (2011a)	Spain	RG-L-L:33(0) CON:32(0)	COM-L-L:42 ± 0 CON:30 ± 0	12	3	Borg (13)	45	COM-L-L CON	FIQ
7	Da Costa et al. (2005)	Canada	AE-M-S:39(0) CON:40(1)	AE-M-S:48 ± 10.0 CON:52 ± 8.0	12	2	73% HRmax	45	AE-M-S CON	FIQ
8	de Almeida Silva et al. (2019)	Brazil	RE-M-S:40(0) CON:25(0)	RE-M-S:44.93 ± 10.30 CON: 49.40 ± 8.30	12	2	70% (1rm)	40	RE-M-S CON	6MWT
9	Ericsson et al. (2016)	Sweden	RE-M-S:56(0) CON:49(0)	22–64	15	2	60% (1rm)	60	RE-M-S CON	FIQ 6MWT
10	Espí-López et al. (2016)	Spain	AE-L-S:13(0) CON:9(0)	AE-L-S:51.2 ± 5.5 CON:57.1 ± 7.1	8	2	Slightly	60	AE-L-S CON	FIQ
11	Ernberg et al. (2018)	Sweden	RE-M-S:49(0) CON:43(0)	RE-M-S:51.2 ± 9.4 CON:48.2 ± 11.4	15	2	75% (1rm)	60	RE-M-S CON	FIQ 6MWT
12	Fontaine and Haaz (2007)	USA	AE-M-S:22(0) CON:26(2)	AE-M-S:48.0 ± 10.0 CON:52.0 ± 8.0	12	3	60% HRmax	30	AE-M-S CON	FIQ Tender point count (1–18) 6MWT
13	Gowans et al. (2001)	Canada	AE-M-S:16(6) CON:15(13)	AE-M-S:49.1 ± 2 CON:46.7 ± 10.3	23	3	68% HRmax	30	AE-M-S CON	FIQ Tender point count (1–18) 6MWT
14	García-Martínez et al. (2012)	Spain	AE-M-S:12(0) CON:13(0)	AE-M-S:59.3 ± 4.8 CON:58.6 ± 7.8	12	3	75% HRmax	60	AE-M-S CON	FIQ
15	Gavi et al. (2014)	Brazil	RE-L-S:35(0) CON:31(0)	RE-L-S:44.34 ± 7.94 CON: 48.65 ± 7.60	16	2	45% (1rm)	45	RE-L-S CON	FIQ
16	Glasgow et al. (2017)	USA	RE-M-S:13(0) CON:12(0)	52 ± 13	8	2	55% HRmax	30	RE-M-S CON	FIQ
17	Häkkinen et al. (2001)	Finland	RE-M-S:11(0) CON:10(0)	RE-M-S:39 ± 6.0 CON:37 ± 5.0	17	2	68% HRmax	60	RE-M-S CON	Tender point count (1–18)
18	Häkkinen et al. (2001)	Finland	RE-M-S:11(0) CON:10(0)	RE-M-S:39 ± 6 CON:37 ± 5	21	2	68% HRmax	60	RE-M-S CON	Tender point count (1–18)
19	Hammond and Freeman (2006)	UK	AE-M-S:71(0) CON:62(0)	AE-M-S:48.36 ± 10.91 CON:48.73 ± 10.95	10	3	68% HRmax	45	AE-M-S CON	FIQ
20	Hernando-Garijo et al. (2021)	Spain	AE-M-S:17(0) CON:17(0)	AE-M-S: 51.81 ± 9.05 CON: 55.06 ± 8.51	15	2	Borg (6)	50	AE-M-S CON	FIQ 6MWT
21	Izquierdo-Alventosa et al. (2020)	Spain	COM-M-S:16(0) CON:16(0)	30–70	8	2	Borg (4)	60	COM-M-S CON	FIQ 6MWT
22	Izquierdo-Alventosa et al. (2021)	Spain	RE-L-S:16(0) con:16(1)	RE-L-S:53.06 ± 8.40 CON:55.13 ± 7.35	15	2	Borg (4)	60	RE-L-S CON	FIQ
23	Jones et al. (2002)	USA	RE-M-S:28(0) CON:28(0)	RE-M-S:49.2 ± 6.36 CON:46.4 ± 8.56	12	2	67% (1rm)	60	RE-M-S CON	FIQ Tender point count (1–18)
24	Jones et al. (2008)	USA	AE-L-S:38(5) CON:38(0)	AE-L-S:45.2 ± 9.4 CON:47.3 ± 7.4	26	3	45% HRmax	60	AE-L-S CON	FIQ Tender point count (1–18)
25	Jablochkova et al. (2019)	Sweden	RE-M-S:38(0) CON:34(0)	50.8 ± 9.6	15	2	75% (1rm)	60	RE-M-S CON	FIQ
26	King et al. (2002)	Canada	AE-M-S:42(0) CON:34(0)	AE-M-S:49.62 ± 7.65 CON:49.78 ± 7.87	12	3	68% HRmax	30	AE-M-S CON	FIQ Tender point count (1–18) 6MWT
27	Kingsley et al. (2005)	USA	RE-M-S:15(0) CON:14(0)	RE-M-S:47 ± 4 CON:45 ± 9	12	2	67% (1rm)	30	RE-M-S CON	FIQ Tender point count (1–18)
28	Kurt et al. (2016)	Turkey	AE-M-S:36(0) CON:36(0)	AE-M-S:35.13 ± 11.60 CON:41.94 ± 12.78	3	3	65% HRmax	35	AE-M-S CON	FIQ
29	Latorre et al. (2013)	Spain	COM-M-L:42(0) CON:30(0)	COM-M-L:52.40 ± 8.01 CON: 50.93 ± 7.72	24	3	74% (1rm)	40	COM-M-L CON	FIQ Tender point count (1–18) 6MWT
30	Latorre Román et al. (2015)	Spain	RE-M-S:20(0) CON:16(0)	RE-M-S:51.70 ± 9.50 CON:50.25 ± 8.83	18	3	74% (1rm)	60	RE-M-S CON	FIQ Tender point count (1–18)
31	Larsson et al. (2015)	Sweden	RE-M-S:67(0) CON:63(0)	RE-M-S:50.81 ± 9.05 CON: 52.10 ± 9.78	15	2	60% (1rm)	60	RE-M-S CON	FIQ 6MWT
32	Munguía-Izquierdo and Legaz-Arrese (2007)	Spain	COM-M-S:29(0) CON:24(0)	COM-M-S:50 ± 7 CON:46 ± 8	16	3	65% HRmax	60	COM-M-S CON	FIQ Tender point count (1–18)
33	Munguía-Izquierdo and Legaz-Arrese (2008)	Spain	COM-M-S:29(0) CON:24(0)	COM-M-S:50 ± 7 CON:46 ± 8	16	3	65% HRmax	60	COM-M-S CON	FIQ Tender point count (1–18)
34	Mannerkorpi et al. (2009)	Sweden	AE-M-S:81(0) CON:85(0)	AE-M-S:44.6 ± 9.26 CON:46.5 ± 8.3	20	1	57% HRmax	45	AE-M-S CON	FIQ 6MWT
35	Paolucci et al. (2015)	Italy	AE-M-S:19(0) CON:18(0)	AE-M-S:50.1 ± 8.9 CON:48.1 ± 10.4	5	2	60% HRmax	60	AE-M-S CON	FIQ
36	Richards and Scott (2002)	UK	AE-L-S:68(0) CON:65(0)	AE-L-S:48 ± 13.3 CON:45 ± 11.9	12	2	Slightly	50	AE-L-S CON	FIQ Tender point count (1–18)
37	Redondo et al. (2004)	Spain	AE-M-S:19(0) CON:21(0)	30–70	8	3	65% HRmax	45	AE-M-S CON	FIQ Tender point count (1–18)
38	Rooks et al. (2007)	USA	AE-M-S:35(0) RE-M-S:135(0) CON:27(0)	AE-M-S:48 ± 11 RE-M-S:50 ± 11 CON:51 ± 12	16	2	50% (1rm)	60	AE-M-S RE-M-S CON	FIQ
39	Rodríguez-Mansilla et al. (2021)	Spain	AE-M-S:33(0) CON:29(1)	52.24 ± 6.19	6	2	65% HRmax	45	AE-M-S CON	FIQ
40	Sañudo et al. (2011)	UK	COM-M-L:18(0) CON:20(0)	COM-M-L:55.48 ± 7.14 CON: 56.15 ± 8.48	24	2	68% HRmax	50	COM-M-L CON	FIQ
41	Sañudo et al. (2012)	Spain	RE-M-L:18(0) CON:19(0)	COM-M-L:55.48 ± 7.14 CON: 56.15 ± 8.48	26	2	68% HRmax	53	COM-M-L CON	FIQ 6MWT
42	Campos et al. (2020)	Spain	COM-M-S:24(0) CON:16(0)	COM-M-S:61.75 ± 8.05 CON: 60.75 ± 7.17	18	3	74% (1rm)	48	COM-M-S CON	FIQ Tender point count (1–18)
43	Tomas-Carus et al. (2007)	Spain	COM-M-S:17(0) CON:17(0)	COM-M-S:51 ± 10 CON:51 ± 9	12	3	70% HRmax	60	COM-M-S CON	FIQ
44	van Eijk-Hustings et al. (2013)	Netherlands	AE-M-S:47(0) CON:48(2)	AE-M-S:43.9 ± 7.6 CON:42.9 ± 11.0	12	2	60% HRmax	55	AE-M-S CON	FIQ
45	Zijlstra et al. (2005)	Netherland	AE-M-S:58(3) CON:76(3)	AE-M-S:48 (22–64) CON: 47 (24–64)	3	2	70% HRmax	60	AE-M-S CON	FIQ Tender point count (1–18)

AE-M-S, Aerobic; Moderate intensity; Short cycle. AE-L-S, Aerobic; Low intensity; Short cycle. AE-L-L, Aerobic; Low intensity; Long cycle. RE-L-S, Resistance; Low intensity; Short cycle. RE-M-S, Resistance; Moderate intensity; Short cycle. COM-L-L, Combined; Low intensity; Short cycle. COM-M-S, Combined; Moderate intensity; Short cycle. COM-M-L, Combined; Moderate intensity; Long cycle. CON, No exercise: control group.

There were 8 types of exercise interventions (AE-L-S: 135 participants, AE-M-S: 540 participants, AE-L-L: 38 participants, RE-L-S: 51 participants, RE-M-S: 400 participants, COM-L-L: 183 participants, COM-M-L: 60 participants, COM-M-S: 49 participants, control group: 1,450 participants). All 45 studies illustrated the cycle of intervention and the number of sessions per week. Further details relating to the interventions are shown in Table 2. In terms of reported outcome measures, 40 studies reported FIQ, and 20 studies reported TPC. There were 13 studies reporting 6WMT as an outcome.

### Results of risk of bias assessment

**Figure 2:**
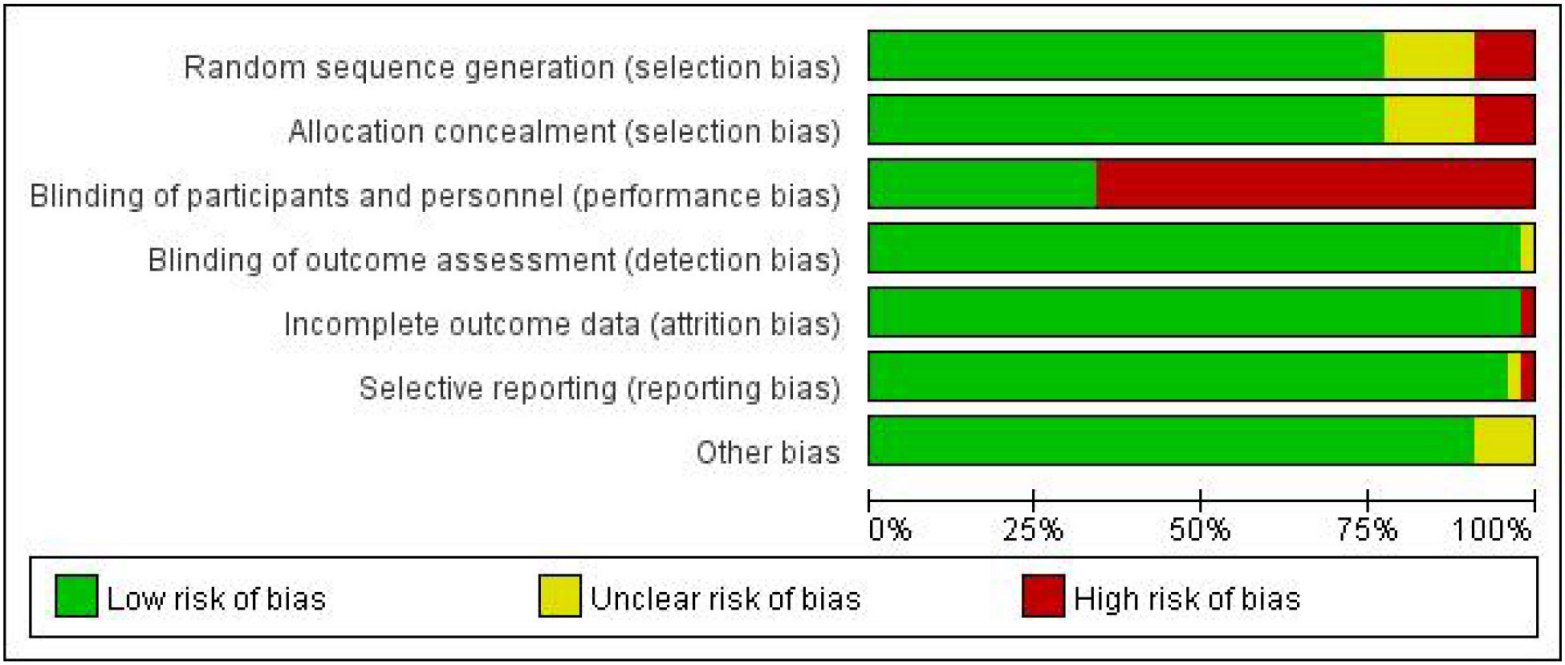
Bias risk of the included studies.

There was a low risk of bias for random sequence generation and allocation concealment in 36 of all 45 studies, four of which were high risk and five of which had an unclear risk of bias. The characteristics of the exercise interventions made blinding participants impossible. Therefore, we assessed “blinded patients and personnel” as “high risk” in 30 studies. Of all 45 studies included, 44 had a low risk of bias for incomplete outcomes, and 44 studies had a low risk of bias for selective outcome reporting. Detailed information about the risks of bias for included studies is shown in Figure 2 (see Supplementary material for details).

### Network meta-analysis

**Figure 3:**
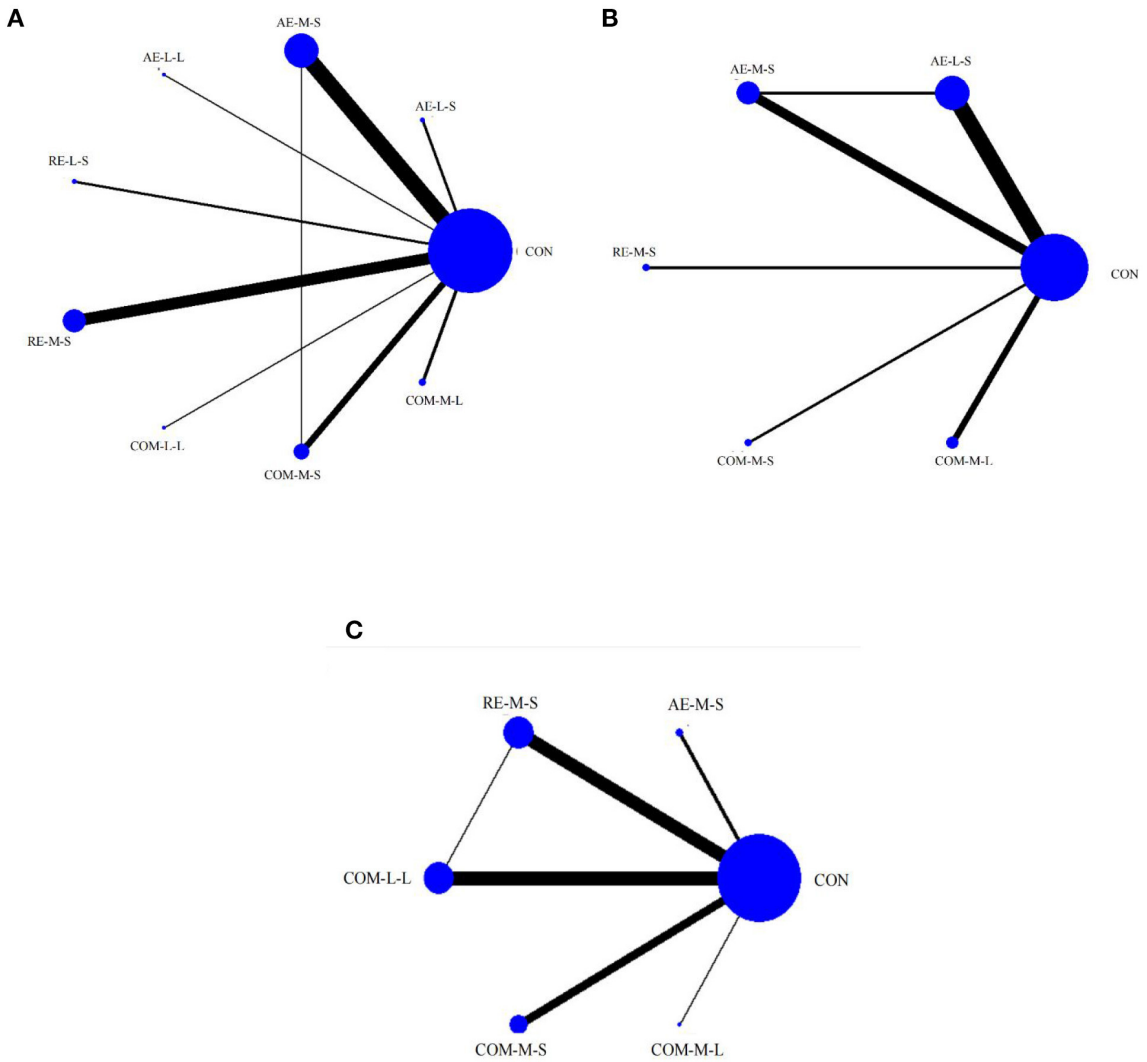
Network meta-analysis maps of the studies examining the efficacy of exercise interventions on **(A)** FIQ, **(B)** TPC, **(C)** 6WMT.

**Figure 4:**
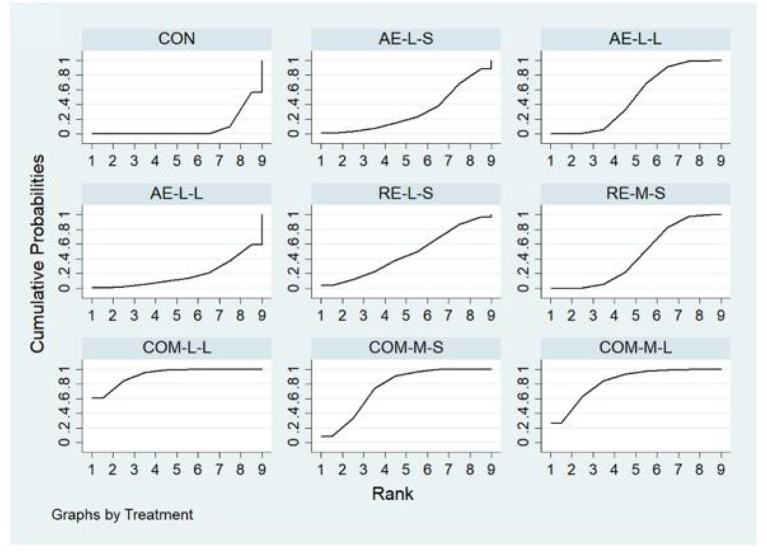
The surface under the cumulative ranking curve (SUCRA) ranking chart for FIQ.

**Figure 5:**
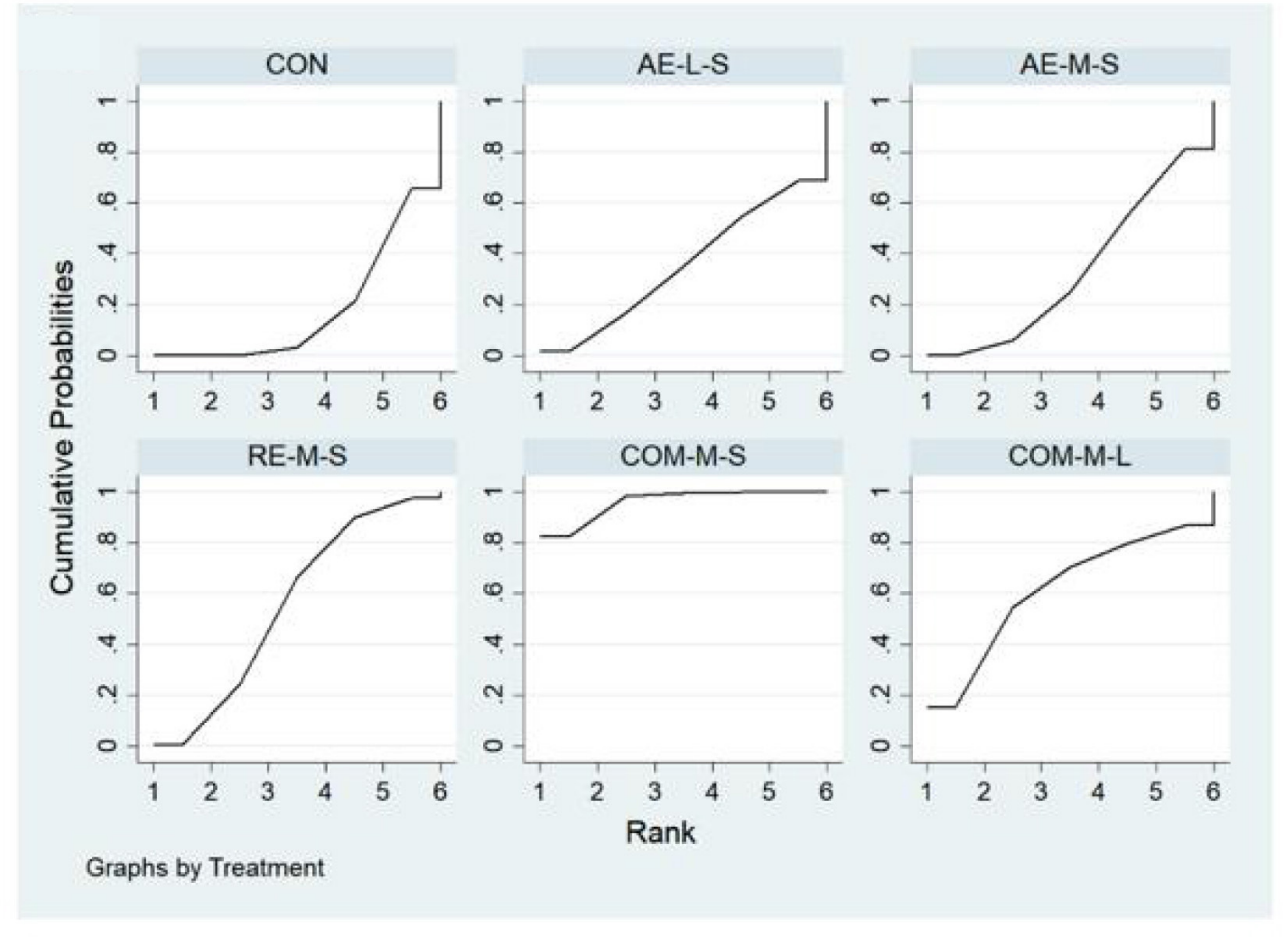
The surface under the cumulative ranking curve (SUCRA) ranking chart for TPC.

**Figure 6:**
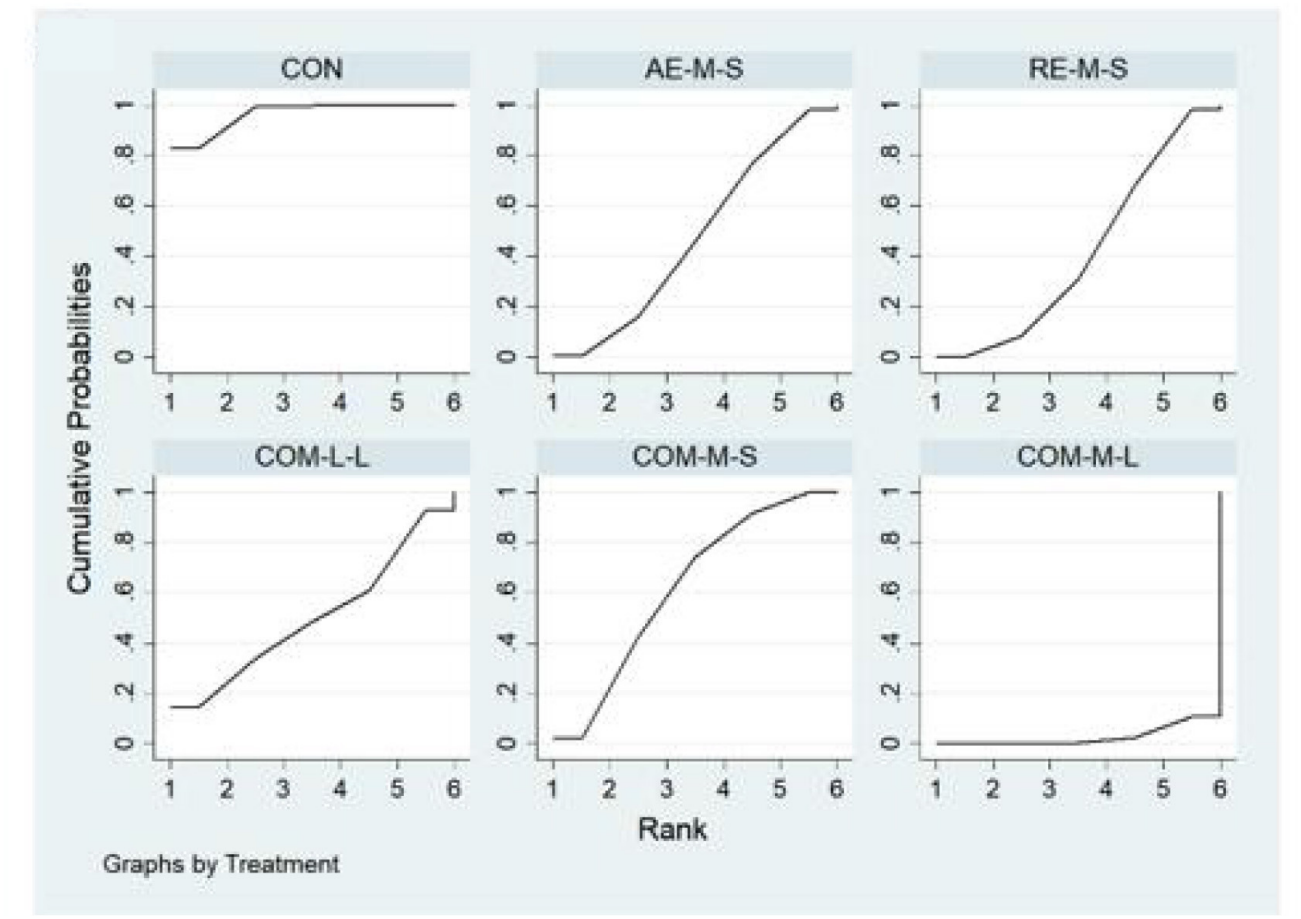
The surface under the cumulative ranking curve (SUCRA) ranking chart for 6WMT.

Measures of quality of life, muscle pain, and physical function for FMS, FIQ, TPC, and 6WMT were all included in the NMA. All networks shared the principles of coherence, transferability, and consistency. Figure 3 shows NMA plots for studies examining the effectiveness of exercise interventions on FIQ, TPC, and 6WMT. The size of the nodes relates to the number of participants for that intervention type and the thickness of the line between interventions relates to the number of studies for that comparison. Tables 3–5 detail the full outcome matrix. Table 6 and Figures 4–6 rank the exercise interventions according to their likelihood of having the desired effect on the outcome being measured.

**Table 3:** Network meta-analysis matrix of results (FIQ).

**Outcome**	**Comparison of treatments: Mean difference (95% confidence intervals) effect of intervention in each row compared** **with intervention in each column**								
**FIQ**									
	CON	COM-M-L	COM-M-S	COM-L-L	RE-M-S	RE-L-S	AE-L-L	AE-M-S	AE-L-S
CON		−11.56 (−17.01, −6.10)	−10.03 (−13.82, −6.25)	−13.40 (−19.08, −7.72)	−6.38 (−9.28, −3.47)			−6.89 (−8.96, −4.83)	
COM-M-L									
COM-M-S		1.53 (−5.11, 8.16)							
COM-L-L		−1.84 (−9.72, 6.03)	−3.37 (−10.19, 3.46)		7.02 (0.64, 13.40)		12.73 (0.98, 24.48)	6.51 (0.47, 12.55)	9.71 (0.16, 19.25)
RE-M-S		5.18 (−1.00, 11.36)	3.65 (−1.12, 8.43)						
RE-L-S	−6.34 (−14.26, 1.57)	5.21 (−4.40, 14.83)	3.69 (−5.09, 12.47)	7.06 (−2.68, 16.80)	0.04 (−8.39, 8.46)				
AE-L-L	−0.67 (−10.95, 9.61)	10.89 (−0.75, 22.52)	9.36 (−1.59, 20.32)		5.71 (−4.98, 16.39)	5.67 (−7.30, 18.65)			
AE-M-S		4.67 (−1.17, 10.50)	3.14 (−1.05, 7.33)		−0.51 (−4.07, 3.05)	−0.55 (−8.72, 7.63)	−6.22 (−16.71, 4.27)		
AE-L-S	−3.69 (−11.36, 3.98)	7.86 (−1.55, 17.27)	6.34 (−2.21, 14.89)		2.68 (−5.52, 10.89)	2.65 (−8.37, 13.67)	−3.02 (−15.85, 9.80)	3.20 (−4.75, 11.14)	

**Table 4:** Network meta-analysis matrix of results (TPC).

**Outcome**	**Comparison of treatments: Mean difference (95% confidence intervals) effect of intervention in each row** **compared with intervention in each column**					
TPC						
	CON	COM-M-L	COM-M-S	RE-M-S	AE-M-S	AE-L-S
CON			−5.09 (−7.62, −2.56)			
COM-M-L	−2.31 (−7.00, 2.38)					
COM-M-S		−2.78 (−8.10, 2.54)		3.39 (0.19, 6.59)	4.57 (1.44, 7.71)	4.51 (0.27, 8.75)
RE-M-S	−1.70 (−3.67, 0.27)	0.61 (−4.47, 5.70)				
AE-M-S	−0.52 (−2.37, 1.33)	1.79 (−3.25, 6.83)		1.18 (−1.31, 3.67)		
AE-L-S	−0.58 (−3.98, 2.83)	1.73 (−4.06, 7.53)		1.12 (−2.82, 5.05)	−0.06 (−3.94, 3.82)	

AE-M-S, Aerobic; Moderate intensity; Short cycle. AE-L-S, Aerobic; Low intensity; Short cycle. AE-L-L, Aerobic; Low intensity; Long cycle. RE-L-S, Resistance; Low intensity; Short cycle. RE-M-S, Resistance; Moderate intensity; Short cycle. COM-L-L, Combined; Low intensity; Short cycle. COM-M-S, Combined; Moderate intensity; Short cycle. COM-M-L, Combined; Moderate intensity; Long cycle. CON, No exercise: control group. The blue color indicates null as the two constructs are compared by themselves.

**Table 5:** Network meta-analysis matrix of results (6WMT).

**Outcome**	**Comparison of treatments: Mean difference (95% confidence intervals) effect of intervention in each row compared with intervention in each column**					
6WMT						
	CON	COM-M-L	COM-M-S	COM-L-L	RE-M-S	AE-M-S
CON		74.80 (38.90, 110.71)			31.80 (12.13, 51.48)	28.79 (6.90, 50.68)
COM-M-L			−54.30 (−95.77, −12.84)			
COM-M-S	20.50 (−0.24, 41.24)					
COM-L-L	27.88 (−24.71, 80.47)	−46.92 (−110.60, 16.76)	7.38 (−49.16, 63.92)			
RE-M-S		−43.00 (−87.18, 1.19)	11.30 (−17.29, 39.89)	3.92 (−52.23, 60.08)		
AE-M-S		−46.01 (−92.60, 0.58)	8.29 (−21.87, 38.45)	0.91 (−56.06, 57.88)	−3.01 (−28.93, 22.90)	

AE-M-S, Aerobic; Moderate intensity; Short cycle. AE-L-S, Aerobic; Low intensity; Short cycle. AE-L-L, Aerobic; Low intensity; Long cycle. RE-L-S, Resistance; Low intensity; Short cycle. RE-M-S, Resistance; Moderate intensity; Short cycle. COM-L-L, Combined; Low intensity; Short cycle. COM-M-S, Combined; Moderate intensity; Short cycle. COM-M-L, Combined; Moderate intensity; Long cycle. CON, No exercise: control group.

**Table 6:** Relative ranking of exercise programs for treatment.

**Outcome**			
**Treatment**	**SUCRA**	**PrBest**	**MeanRank**
**FIQ**			
CON	8.4	0.0	8.3
AE-L-S	30.6	0.8	6.6
AE-M-S	49.8	0.0	5.0
AE-L-L	18.6	0.9	7.5
RE-L-S	47.2	3.6	5.2
RE-M-S	45.1	0.0	5.4
COM-L-L	92.3	60.7	1.6
COM-M-S	75.2	8.0	3.0
COM-M-L	82.8	26.0	2.4
**TPC**			
CON	17.0	0.0	5.1
AE-L-S	35.8	1.4	4.2
AE-M-S	32.4	0.1	4.4
RE-M-S	58.0	1.2	3.1
COM-M-S	96.1	82.1	1.2
COM-M-L	60.7	15.1	3.0
**6WMT**			
CON	96.5	82.9	1.2
AE-M-S	47.4	0.3	3.6
RE-M-S	41.1	0.0	3.9
COM-L-L	50.2	14.5	3.5
COM-M-S	62.1	2.3	2.9
COM-M-L	2.8	0.0	5.9

AE-M-S, Aerobic; Moderate intensity; Short cycle. AE-L-S, Aerobic; Low intensity; Short cycle. AE-L-L, Aerobic; Low intensity; Long cycle. RE-L-S, Resistance; Low intensity; Short cycle. RE-M-S, Resistance; Moderate intensity; Short cycle. COM-L-L, Combined; Low intensity; Short cycle. COM-M-S, Combined; Moderate intensity; Short cycle. COM-M-L, Combined; Moderate intensity; Long cycle. CON, No exercise: control group.

#### FIQ

**Figure 7:**
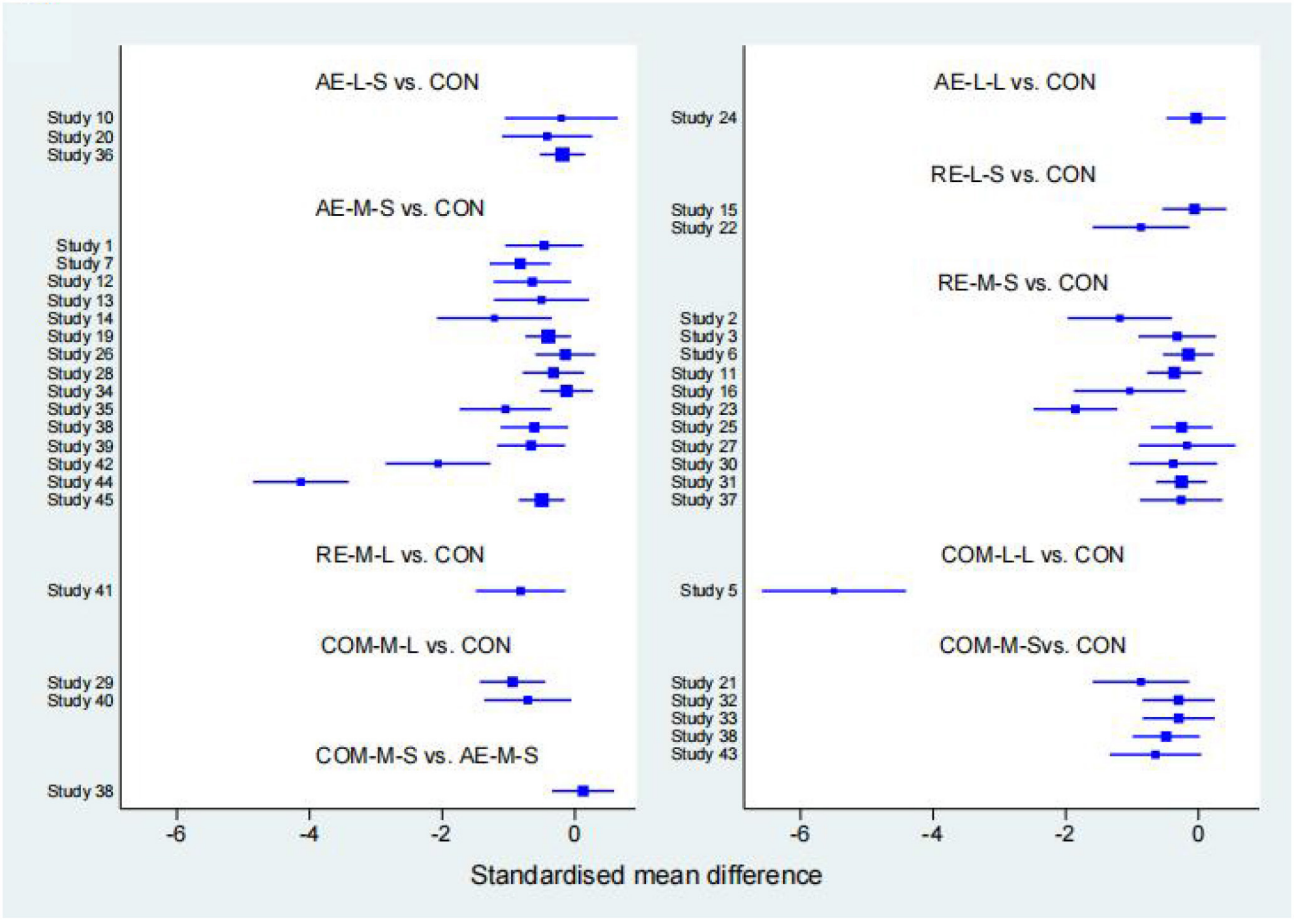
The forest map for FIQ.

Forty studies, with 2,454 participants and 8 intervention categories contributed to the NMA assessing FIQ (see Figure 7 for a forest plot). Aerobic exercise (AE-M-S, AE-L-S, and AE-L-L) contributed 23.8% of the data, resistance exercise (RE-L-S, RE-M-S) 17.5%, combined exercise (COM-L-L, COM-M-S, and COM-M-L) 11%, and the control the remaining 48.8%. Overall, interventions that included an aerobic exercise [AE-M-S: −6.89 [CI = −8.96, −4.83]], resistance-exercise {RE-M-S: −6.38 [CI = (−9.28, −3.47)]} combined-exercise {COM-L-L: −13.40 [CI = (−19.08, −7.72)]; COM-M-S: −10.03 [CI = (−13.82, −6.25)]; COM-M-L: −11.56 [CI = (−17.01, −6.10)]} were effective in decreasing FIQ against control group (Table 3). Table 6 illustrates the average ranking of exercise interventions according to the likelihood of affecting FIQ. Considering direct and indirect comparisons, COM-L-L had the highest likelihood of improving FIQ.

#### TPC

**Figure 8:**
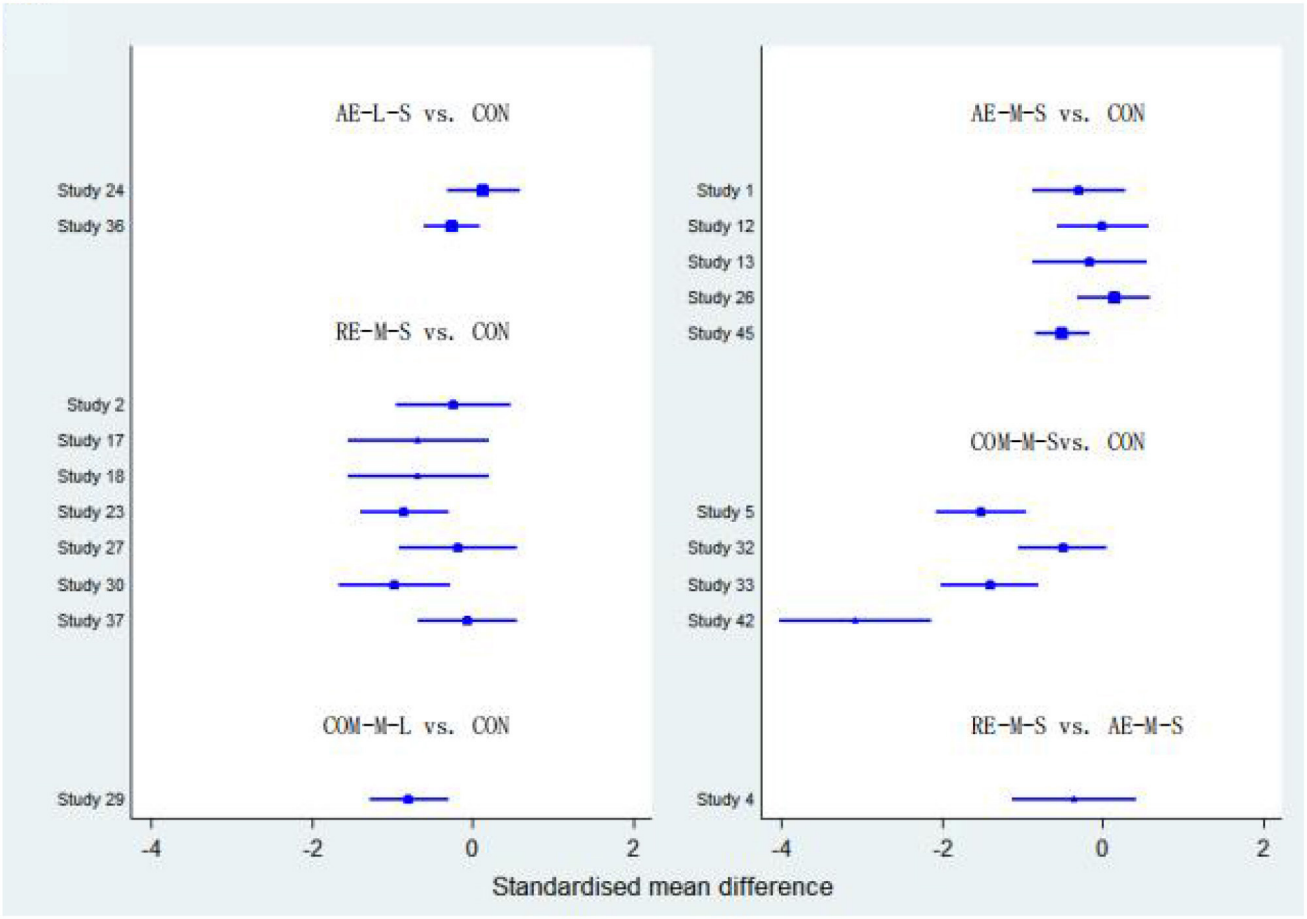
The forest map for TPC.

Twenty studies, including 1,085 participants and all 5 exercise intervention categories, contributed to this analysis (see Figure 8 for a forest plot). Most of the data analyzed came from aerobic interventions (22.5%). Resistance and combined exercise contributed 17.5 and 12.5%, respectively. The control group accounted for the remainder (47.5%). Only the exercise intervention combining aerobic and resistance exercise [COM-M-S: −5.09 [CI = −7.62, −2.56]] was found to be significantly effective in reducing TPC against the control group. Table 6 shows the mean ranking of the exercise interventions according to the likelihood of affecting TPC.

#### 6WMT

**Figure 9:**
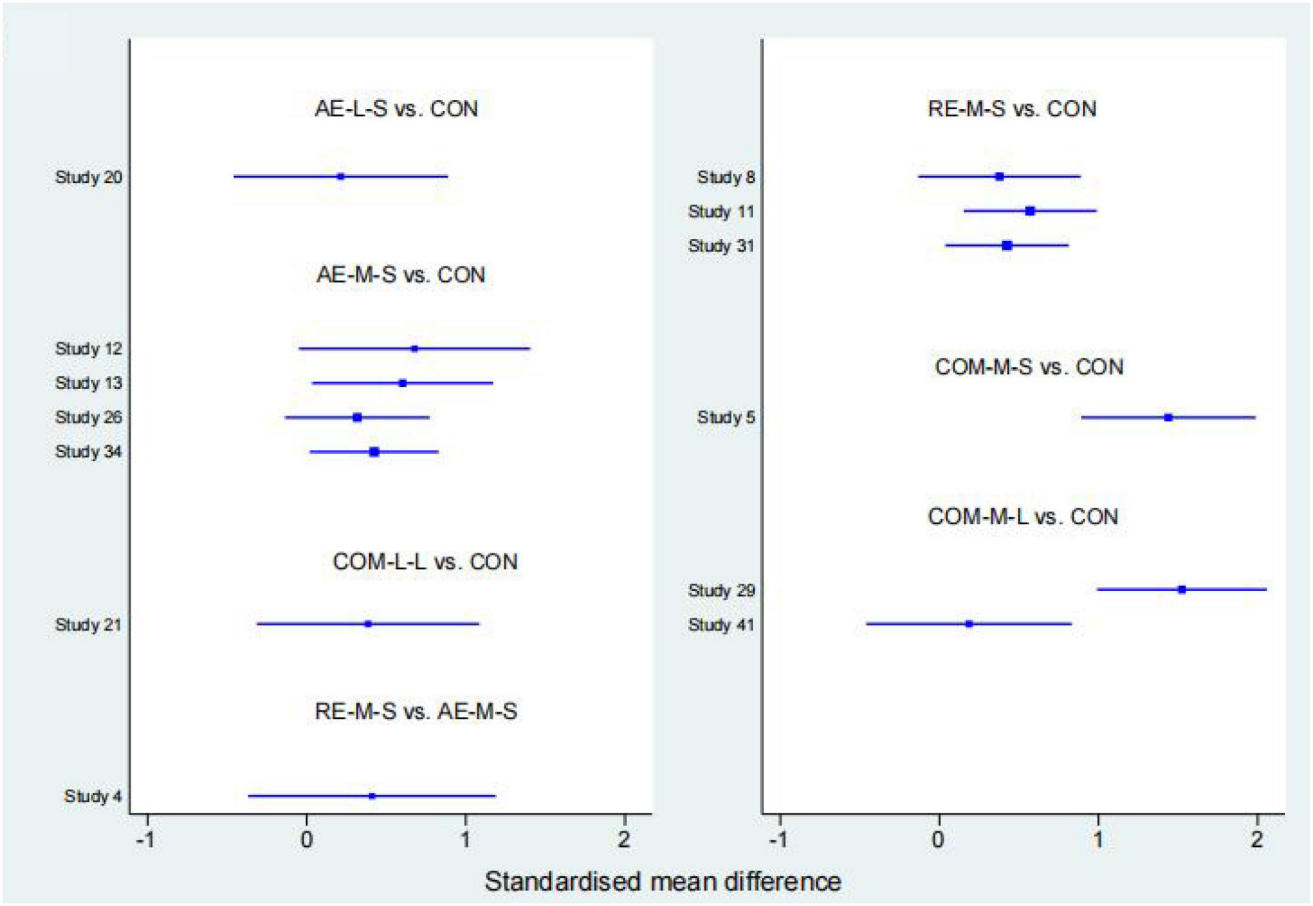
The forest map for 6WMT.

Thirteen studies, including 776 participants and all 5 exercise intervention categories (AE-M-S, RE-M-S, COM-M-L, COM-M-S, COM-L-L) contributed to this analysis (see Figure 9 for a forest plot). Most of the data analyzed came from aerobic interventions (23.1%). Resistance and combined exercise contributed 15.4 and 11.5%, respectively. The control group accounted for the remainder (50%). Of these, three exercise interventions [COM-M-L: 74.80 [CI = 38.90, 110.71]; RE-M-S: 31.80 [CI = 12.13, 51.48]; AE-M-S: 28.79 [CI = 6.90, 50.68]] were found to be significantly effective in improving 6WMT against control group. By taking into account direct and indirect comparisons. COM-M-L was most likely to improve 6WMT.

## Discussion

This NMA combined direct and indirect evidence from 45 RCTs of exercise interventions for patients with fibromyalgia syndrome and compared 8 different interventions for over 2,400 patients having fibromyalgia syndrome. Our main findings suggested that aerobic exercise (AE-M-S), resistance exercise (RE-M-S, RE-M-L), and combined exercise (COM-L-L, COM-M-S, COM-M-L) were all significantly effective in improving the quality of life in patients with fibromyalgia syndrome. In terms of relieving muscle pain in FMS, only combined exercise (COM-M-S) produced a significant positive effect. In addition, aerobic exercise (AE-M-S), resistance exercise (RE-M-S), and combined exercise (COM-M-L) were found to significantly improve physical function in FMS. Therefore, a combination of the exercises was the most promising exercise program to improve quality of life, relieve muscle pain, and enhance physical function in FMS at the same time.

The noticeable and common negative impact of fibromyalgia syndrome was a reduction in quality of life, mainly in the form of poor work performance, impoverished sleep quality, and increased levels of depression and anxiety (Larsson et al., 2015). The Fibromyalgia Impact Questionnaire (FIQ) was widely used to measure the quality-of-life measure for FMS. The FIQ contains pain, fatigue, stiffness, anxiety, and depression, with lower scores indicating better quality of life (Williams and Arnold, 2011). For a long time, aerobic exercise and resistance exercise have been the most common exercise programs. The two programs have been consistently shown to be effective in relieving symptoms of depression and fatigue in patients with FMS, with no adverse events associated with exercise (Bidonde et al., 2017). In addition, the European League Against Rheumatism (EULAR) recommends aerobic exercise and strength exercise in individualized exercise programs for the treatment of patients with fibromyalgia (Carville et al., 2008). Therefore, the two exercise programs are considered to be effective in sounding the symptoms associated with patients with fibromyalgia syndrome. However, previous studies have found that aerobic or resistance exercise alone can not completely overcome all the negative effects of fibromyalgia syndrome. For example, Bidonde et al. (2017) systematically evaluated 13 studies on the efficacy of aerobic exercise interventions for FMS and found low-intensity evidence that aerobic exercise interventions were effective in improving patients' pain but not fatigue and stiffness. To resolve the problems, an increasing number of researchers have introduced a combination of the two into the treatment of fibromyalgia syndrome in recent years, and results showed that the combination of aerobic and resistance exercises could achieve or even surpass the effects of the first two exercise programs alone (Campos et al., 2020; Izquierdo-Alventosa et al., 2020). These results were consistent with the results of the present study. In this study, we found that although aerobic exercise (AE-M-S), resistance exercise (RE-M-S, RE-M-L), and combined exercise (COM-L-L, COM-M-S, COM-M-L) all significantly improved the quality of life in FMS, the mean ranking of exercise interventions according to their likelihood of affecting FIQ showed that combined exercise (COM-L-L) had the greatest likelihood of improving FIQ, and for patients with fibromyalgia syndrome, a low-intensity, long-period exercise program is more likely to be accepted and adhered to until the benefits of exercise are achieved. Interestingly, combined exercise was better than aerobic or resistance exercise in the same conditions that included exercise intensity, exercise frequency, exercise duration, and intervention period. It is therefore reasonable to assume that exercise combined with aerobic and resistance exercise can help to improve quality of life and general health.

Fibromyalgia is a condition characterized by persistent widespread pain and pressure points (Jones et al., 2008), and pain has been recognized by the American College of Rheumatology (ACR) as a core symptom of FMS. Since the 1990s, in the ACR-approved classification criteria for fibromyalgia, the ”American College of Rheumatology 1990 Classification Criteria for Fibromyalgia,” and the formally proposed criteria include pressure points (Wolfe et al., 1990). Previous studies have demonstrated that aerobic exercise, resistance exercise, and aerobic combined with resistance exercise can be effective in reducing pain symptoms in patients (Espí-López et al., 2016; Andrade et al., 2019). For FMS, increasing physical activity through a designated exercise program will significantly decrease pain, dysfunction, and disability (Bidonde et al., 2014). For example, traditional Chinese exercises such as Tai Chi, Qi Gong, and Ba Duan Jin are very popular among the elderly population, with Tai Chi and Qi Gong being found to be effective in alleviating pain in FMS (Lynch et al., 2012; Wang et al., 2018). Resistance exercise with Swiss balls and dumbbells has also been shown to have a similar effect on reducing FMS pain (Tomas-Carus et al., 2009; Latorre Román et al., 2015). Although this study does not have strong evidence to consolidate the above two views, the present study found that the comprehensive exercise also has a positive effect on reducing the pain symptoms of FMS, which are consistent with previous studies (Gusi et al., 2006; Paolucci et al., 2015).

Although low levels of physical functioning are a common manifestation of FMS, they are often easily disregarded and the consequences can be dire (Shaver et al., 2006; Rutledge et al., 2007). On the one hand, low levels of physical functioning deteriorate with age, causing great inconvenience to the lives of FMS (Geneen et al., 2017). On the other hand, it can be accompanied by a greater risk of disability and financial burden, ultimately putting patients at risk of losing their independence (Shillam et al., 2011). Previous studies have found that both aerobic exercise and resistance training are effective in improving patients' levels of physical function, but less attention has been paid to combined exercise. The present study found that the exercise program incorporating resistance exercise was promising in terms of physical function gains, This finding may be related to the mechanism of resistance exercise for FMS: resistance exercise enables a significant increase in muscle strength in FMS, effectively enhancing the skeletal muscle strength of FMS patients, thereby reducing their skeletal muscle dysfunction and improving their level of physical function (Busch et al., 2013). While aerobic exercise focuses on FMS cardiorespiratory endurance exercise, this exercise alone is not effective in improving skeletal muscle strength in FMS. The combination of resistance exercise, it can enable FMS to make greater gains in aerobic exercise. The results of the present study supported this view by highlighting the value of the joint effects of two exercises. In conclusion, people who wish to improve physical function in FMS through exercise program need to choose the types of exercise carefully.

In terms of the overall contribution of the present study, it was clear that (1) the review was systematic and exhaustive. A sizeable sample of FMS was included (*n* = 2,409), thus providing the ability to detect statistically significant mean differences. (2) The included literature was all randomized controlled trials (RCTs). (3) This was the first time that exercise therapy was assessed comprehensively in terms of three dimensions: quality of life, pain, and physical function, as few fibromyalgia studies assessing exercise interventions used all three dimensions together to evaluate the efficacy of exercise.

At the same time, the current NMA has several limitations: (1) Although there were 8 exercise interventions included in the study, the number of people in each exercise program varied dramatically. Accordingly, the proportions were not sufficiently coordinated and were prone to error. (2) There were many indicators of pain and physical function that can reflect FMS, such as SF-36, VAS, Hand grip force, Elbow flexion force, Knee extension force, etc. Although Tender point count and 6WMT can reflect the pain and physical function of FMS to a great extent, the selection of outcome indicators was still limited and might lead to biased results. Future studies should include as many compatible outcome indicators as possible. (3) Due to the specific nature of fibromyalgia, the gender difference between the patients included in the study was too large. The present study focused less on male patients. Future studies could focus on exercise therapy for the male population. (4) This study did not take into account the volume of training. For example, a resistance training session can consist of 3 × 10 reps (5 exercises) and another with 3 × 20 reps (10 exercises), with the same intensity. This would lead to a different training load and necessarily different adaptations. This would possibly lead to a different training load and necessarily different adaptations. In future studies, additionally, the length of training sessions could also be included, not just the cycle length.

Despite the above limitations, this NMA provided valuable information on the clinical application of exercise interventions in the management of fibromyalgia. The results of this review showed that in FMS, the best exercise protocol to generate the most clinically meaningful change in FIQ was the COM-L-L. The best exercise protocol to achieve above goal in TPC was the COM-M-S. The best exercise program to induce the most clinically significant change in 6WMT was the COM-M-L. The type of exercise that improves both the quality of life, pain, and physical function in FMS was a combination of exercises.

## Data availability statement

The original contributions presented in the study are included in the article/Supplementary material, further inquiries can be directed to the corresponding author.

## Author contributions

JC and CW: conceptualization and writing—review and editing. JC: methodology, formal analysis, writing—original draft preparation, and visualization. JC and BH: validation and data curation. CW: supervision. All authors have read and agreed to the published version of the manuscript.

## Funding

The study was supported by Shanghai Philosophy and Social Science Planning Project (2020EYY004), Innovative Research Team of Shanghai International Studies University (2020114052), and Shanghai Chenguang Talent Program (20CG40).

## Conflict of interest

The authors declare that the research was conducted in the absence of any commercial or financial relationships that could be construed as a potential conflict of interest.

## Publisher's note

All claims expressed in this article are solely those of the authors and do not necessarily represent those of their affiliated organizations, or those of the publisher, the editors and the reviewers. Any product that may be evaluated in this article, or claim that may be made by its manufacturer, is not guaranteed or endorsed by the publisher.
