# Supplementary figures and images for: On the superiority of a combination of aerobic and resistance exercise for fibromyalgia syndrome: A network meta-analysis
Source: Front Psychol. 2022 Sep 28;13:949256. doi: 10.3389/fpsyg.2022.949256 (PMC9554347; doi:10.3389/fpsyg.2022.949256)

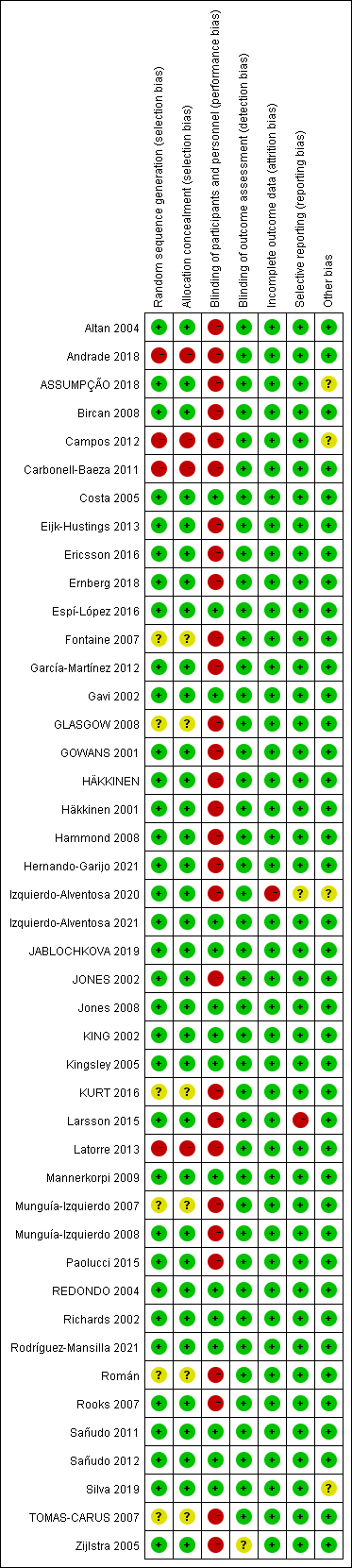


**Figure S-1** **Bias risk of the included studies (details)**

Supplement: Supplementary file 1 [file Table_1.DOCX]
